# The role of IL-1 in postprandial fatigue

**DOI:** 10.1016/j.molmet.2018.04.001

**Published:** 2018-04-12

**Authors:** Louise L. Lehrskov, Emma Dorph, Andrea M. Widmer, Matthias Hepprich, Judith Siegenthaler, Katharina Timper, Marc Y. Donath

**Affiliations:** 1 The Centre of Inflammation and Metabolism and the Centre for Physical Activity Research, Rigshospitalet, Rigshospitalet 7641, Blegdamsvej 9, DK 2100, Copenhagen, Denmark; 2 Clinic of Endocrinology, Diabetes & Metabolism and Department Biomedicine, University Hospital Basel, Spitalstrasse 21, 4031, Basel, Switzerland

**Keywords:** Postprandial fatigue, Interleukin-1, Anakinra and obesity

## Abstract

**Objectives:**

Cytokines such as IL-1 seems to play a role in the pathogenesis of fatigue associated with some chronic diseases and anti-inflammatory treatment has been shown to reduce these symptoms.

Ingestion of a calorie rich meal leads to postprandial fatigue, and is associated with increased systemic concentrations of cytokines, which is more pronounced in obese than lean subjects.

We investigated whether postprandial fatigue is regulated by IL-1, and therefore reduced by IL-1 antagonism, in lean and obese subjects.

**Methods:**

In a double-blind, crossover study in 8 lean and 8 obese male subjects, randomized to receive either saline (placebo) or the IL-1 receptor antagonist anakinra, we investigated whether postprandial fatigue was regulated by IL-1. To promote postprandial fatigue, subjects ran 30 min prior to a high-fat, high-carbohydrate meal. Fatigue was determined using the Stanford Sleepiness Scale and blood samples were drawn at baseline and after the intervention.

**Results:**

IL-1 antagonism led to a reduction in postprandial fatigue and this effect was more pronounced in obese than lean individuals.

**Conclusions:**

We conclude that IL-1 is involved in the regulation of postprandial fatigue under physiologic conditions in lean and obese individuals. It remains to be shown whether this effect translates into clinical relevant effects.

## Highlights

• Ingestion of a calorie rich meal leads to postprandial fatigue
• IL-1 contributes to postprandial fatigue in lean and obese individuals.
• IL-1 antagonism may decrease postprandial fatigue in humans.

## Graphical abstract

**Figure:**
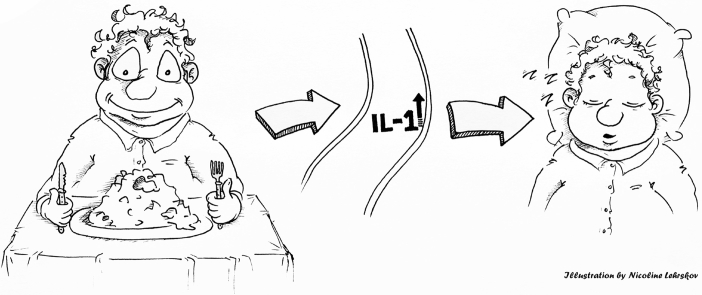

## 1 Introduction

Perception of fatigue has been linked to interleukin-1 (IL-1) – a family of 11 cytokines playing an important role in the initiation and regulation of the inflammatory response [1].

IL-1α and IL-1β have been described most frequently in the literature on fatigue [2]. IL-1α, IL-1β, and IL-1 receptor antagonist (IL-1Ra) bind to the IL-1 receptor. Whereas IL-1α and IL-1β activate the receptor and thereby the inflammatory signal, IL-1Ra has the opposite effect and inhibits the inflammatory response [1].

Chronic Fatigue Syndrome (CFS) is a medically unexplained syndrome characterized by severe disabling fatigue over a period of at least 6 months [3]. Chronic fatigue is associated with an increased level of proinflammatory cytokines such as IL-1α and IL-1β, which has been speculated to be driving fatigue [4].

The perception of fatigue is furthermore commonly reported in a variety of other inflammatory diseases such as multiple sclerosis [5], rheumatic arthritis [6], cancer [7], [8], diabetes [9], metabolic syndrome [10], and also obesity [11]. These diseases all share features of chronic inflammation with an increased level of IL-1α and IL-1β and other cytokines [1], [2], [12], [13], [14].

The pathogenesis leading to increased perception of fatigue in inflammatory diseases is not fully understood, but IL-1 has been suggested to play a role [2], [4], as an increased level in the brain leads to increased perception of fatigue [15], [16]. IL-1α and IL-1β like other cytokines are able to reach and act on the brain in different ways, and the IL-1 receptor is distributed throughout the brain. So even though IL-1 cytokines are primarily produced in the periphery, they have the ability to affect the central nervous system responsible for the perception of fatigue [4], [17].

Further supporting the role of IL-1 cytokines in the perception of fatigue comes from studies where blocking of IL-1 with anakinra (recombinant IL-1Ra) [9] and other IL-1 blocking agents reduced the perception of fatigue [2].

Experiencing postprandial fatigue and poor mental performance is common after ingestion of a large meal [18], [19], [20], [21]. Intraduodenal lipid infusions resulted in reduced alertness and accuracy in attention tasks compared to saline infusions [22]. The source of calories seems to play a role as alertness was found to be lower after ingestion of a high fat/low carbohydrate meal compared to a low fat/high carbohydrate meal [22], [23].

The underlying mechanisms leading to postprandial fatigue are not well understood. Along with postprandial fatigue and poor mental performance, circulating concentrations of cytokines increase in response to a meal [24], [25], [26]. Fat is a strong inducer of cytokines, however, a high fat containing meal combined with a carbohydrate rich drink leads to an even more pronounced increase [27]. The literature provides inconsistent information regarding the increase of IL-1β in response to a meal. Recently, we have shown that postprandial IL-1β increases in response to a meal in mice [28], and in humans increased plasma IL-1β was found after intake of a high fat meal [29] whereas others show no effect of high fat intake on IL-1β [30], [31], [32], which is possibly due to the complexity in measuring circulating cytokines [33]. Furthermore, plasma levels of IL-1 is often only slightly elevated in plasma even during severe pathology [34], as a large part of IL-1 is located within leukocytes [1]. Finally, postprandial elevation in inflammatory cytokines has been shown to be greater in obese subject [12], [13].

Taken together, pathological fatigue is associated with an elevation in circulating proinflammatory cytokines, which can be partly inhibited by IL-1 antagonism. Because the IL-1 system is activated following food ingestion, we hypothesized that also postprandial fatigue is regulated by IL-1 and can be reduced by IL-1 antagonism. Moreover, obesity leads to chronic low-grade inflammation and an increased postprandial cytokine response, which could possibly lead to more pronounced postprandial fatigue. Thus, we included a cohort of both lean and obese individuals to investigate the role of IL-1 in postprandial fatigue.

## 2 Experimental procedures

### 2.1 Subjects and screening

Healthy male subjects between 18 and 65 years old, who usually ate breakfast and lunch, were included in the study. 8 subjects were lean (BMI > 18 and ≤ 28 kg/m^2^) and 8 subjects were obese (BMI > 30 and ≤ 38 kg/m^2^). Exclusion criteria were smoking, night-shift work, sleep disturbances, clinical signs of infection, diabetes, haematologic, renal, hepatic, cardiac, pulmonary or inflammatory disease, history of carcinoma or tuberculosis, increased alcohol consumption or known allergy to anakinra, paracetamol or ingredients in the test meals. Further, subjects were excluded if they had current treatment with any drugs including vitamin supplementation or had used the investigational drug within 30 days prior to enrolment or within 5 half-lives of the investigational drug, whichever was longer. Although smoking was an exclusion criterion, we included one smoker, as he was willing to pause smoking 24 h prior to the study days.

### 2.2 Study design

The study was a randomized placebo-controlled, double blind, crossover study investigating the effect of IL-1 antagonism on postprandial fatigue symptoms in lean and obese but otherwise healthy subjects.

Recruitment of subjects and both study visits were performed from August 2016 until April 2017 at the University Hospital in Basel, Switzerland. All procedures are in accordance with ICH-GCP guidelines and Declaration of Helsinki. The study was approved by the Ethics Committee in Basel (EKNZ BASEC2016-00816) and registered on clinicaltrials.gov (NCT02916355).

All subjects gave oral and written consent prior to participation in the study.

### 2.3 Treatment assignment and blinding

Once screening was completed and eligibility was confirmed, subjects were randomly assigned to receive placebo (saline) or the study medication, anakinra. The Clinical Trial Unit at the University Hospital in Basel, Switzerland, was responsible for treatment blinding, preparation and injection of study medication. Subjects as well as the investigators were blinded throughout the study.

### 2.4 Fatigue measurement

The Stanford Sleepiness Scale (SSS), developed by Hoddes et al. [35], is a validated method used to measure subjective sleepiness [36]. The Stanford sleepiness scale consists of eight levels (Table A.1, Appendix) and subjects are asked to indicate which level described their current state best.

The Epworth Sleepiness Scale (ESS) (Table A.2, Appendix), developed by Johns [37], is a self-administrated scaled used to measure general level of daytime sleepiness. The Epworth Sleepiness Scale tries to encounter the different routines in people's daily life [36]. The Epworth Sleepiness Scale consists of eight common everyday situations, which the subject rates on a scale from 0 to 3, describing how quickly they would fall asleep or doze off in the given situation.

The total score is between 0 and 24 and a score of 10 or higher indicates abnormal or pathological sleepiness.

To ensure that sleep prior to the study visits did not influence the outcome on study days, subjects recorded their sleep for three nights prior to the study visits. This was preferably done using a smartphone app (Sleep Cycle), available in mobile applications (apps) stores, which subjects were asked to download on the screening visit. Not all subjects managed to use Sleep Cycle, therefore some subjects gave an estimate of how many hours they had slept and how many times they were awake during the night.

### 2.5 Anakinra

All subjects received anakinra on one of the two study days. Anakinra (Swedish Orphan Biovitrum) is a recombinant human IL-1Ra. Anakinra has a half-life of 4–6 h, and maximal plasma concentration is reached 3–7 h after administration. It is administrated by subcutaneous injection (0.67 ml) and the most common side effect is local skin reaction at the injection-site, however, this does only occur after repetitive injections over several days.

### 2.6 Study procedure

The study consisted of one screening visit followed by two study visits separated by seven days each. All subjects went through a medical screening, including a blood chemistry screen and an electrocardiogram.

Subjects received a standardized dinner prior to each study days. The standardized dinner consisted of 5 cheese pies (15 g fat, 25 g carbohydrates and 8 g protein pr. 100 g) and an apple and a banana, a total of 1100 kcal. The cheese pies and fruit were acquired from the supermarket ‘COOP’. The meal was to the subjects on the screening visit. The rationale behind this choice of meal was that it was easy to prepare, and the amount of energy and composition of macronutrients of the meal was within the range of what we would expect the subjects to eat for a regular dinner [38], [39].

The evening prior to each study day, subjects were allowed to eat solely a standardized dinner with water and then they fasted, for at least 10 h, before arriving in the study center at 8 o'clock. Afterwards, a short medical examination was performed. Subjects filled in the Epworth Sleeping Scale and Stanford Sleepiness Scale and afterwards a subcutaneous injection of placebo (saline) or 0.67 ml anakinra was performed.

In order to enhance postprandial fatigue, we exposed the subjects to 30 min of running at 75% of maximum heart rate. Heart rate was estimated using the formula; 220 – Age.

Heart rate was measured during the 30 min run and through the following 6 h using a heart rate monitor. After the 30 min exercise bout the Stanford Sleepiness Scale was filled in and an intravenous catheter was placed in the forearm for later blood sampling. The Stanford Sleepiness Scale was filled in before receiving the study meal and throughout the study day (time point −3, −1, 0, 1, 1.5, 2, 3, 3.5 and 4 h). Blood samples for inflammatory parameters (CRP and IL-6) were drawn pre and post intervention (time point – 3 and 3 h).

**Figure 1:**
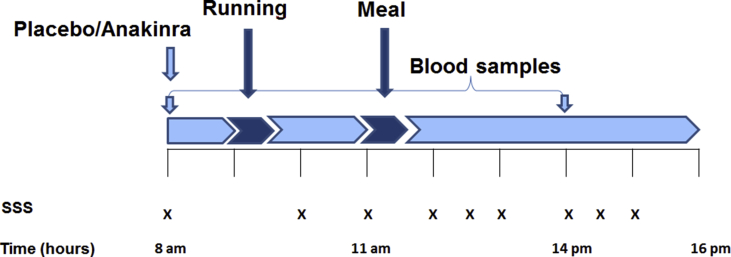
**Study days**. Outline of the study visits. Subjects were running at 9 am for 30 min at 75% of maximum heart rate. The study meal was served at 11 am and subjects had 30 min to consume the meal. The Stanford Sleepiness Scale were used regularly (X) throughout the study days.

The study meal was served at time point 0 on both study days and consumed within 30 min. The meals were identical on both study days and contained a total of 1404 kcal. See Table A.3 (Appendix) for details in nutritional composition. A study overview is presented in Figure 1.

### 2.7 Study endpoints

The primary outcome was the difference in fatigue between groups treated with IL-1Ra (anakinra) vs. placebo (saline) measured by the Stanford Sleepiness Scale. The secondary outcomes were postprandial changes in plasma levels of inflammatory markers (IL-6 and CRP) due to any treatment anakinra vs. placebo.

### 2.8 Sample collection and analytic procedure

Measure of CRP, IL-6, HbA1c, glucose, and insulin were performed by automated biochemical analyses in the University Hospital Central Laboratories in Basel.

CRP: Latex particle-based immunoassay (LIA) – turbidimetry. Machine: Cobas 8000, c702 module. IL-6: Sandwich Elektrochemiluminescens immunoassay (ECLIA) – photon counting. Machine Cobas 8000, e602 module. Glucose: Enzymatic determination – absorption spectrophotometry. Machine: Cobas 8000, c702 modul. Insulin: Sandwich Elektrochemiluminescens immunoassay (ECLIA) – photon counting. Machine Cobas 8000, e602 module.

HbA1c: Liquid chromatography (LC) – absorption spectrophotometry. Machine: Tosoh G7, Tosoh G8.

### 2.9 Statistical analysis

No formal power calculation was performed, as this study, to the best of our knowledge, is the first of its kind. Results are expressed as means ± SEM, and significance was accepted with *p* < 0.05. Statistical analyses were performed using GraphPad Prism version 7.02 (GraphPad Software, La Jolla, California, USA). Student's paired t-test was used to analyse SSS data in all 3 groups (lean, obese and when lean and obese subjects were grouped) comparing saline vs. IL-1Ra at specific time points.

Epworth Sleepiness Scale, hours slept prior to study visits and inflammation markers (CRP and IL-6) were compared using Student's paired t-test when comparing saline vs. IL-1Ra, and an unpaired t-test when comparing lean vs. obese.

## 3 Results

### 3.1 Participants flow

18 subjects were enrolled in the study and 16 completed the study. Two subjects dropped out before the first study visit, one due to sickness and one was unable to participate on the given days. Thus, in total, 8 lean subjects and 8 obese subjects went through the entire study procedure. The baseline characteristics of the participants are presented in Table 1.

**Table 1:** **Baseline characteristics of participants**. Data represent the mean ± SEM.

Characteristics	Lean (n = 8)	Obese (n = 8)	P-value
Age (years)	28.8 ± 3.4	31.0 ± 2.9	0.62
Weight (kg)	73.6 ± 4.5	111.8 ± 4.8	<0.0001
Body Mass Index (kg/m^2^)	23.8 ± 1.1	32.7 ± 1.0	<0.0001
Blood pressure systolic (mmHg)	127 ± 4	130.5 ± 3.1	0.44
Blood pressure diastolic (mmHg)	78 ± 2	82.9 ± 2.1	0.11
CRP (mg/l)	1.1 ± 0.3	1.5 ± 0.4	0.35
HbA1c (mmol/mol)	5.3 ± 0.2	5.6 ± 0.2	0.25
Fasting blood glucose (mmol/l)	4.8 ± 0.2	6.1 ± 0.7	0.12
Insulin (pmol/l)	6.2 ± 0.8	18.1 ± 4.2	0.02

### 3.2 Sleep registration prior to study visits and Epworth Sleepiness Scale

To monitor potential variation in sleeping pattern before the two study days, subjects were asked to record the hours slept three nights prior to each study visit and to answer the Epworth Sleepiness Scale on each study visit [37]. Data showed no difference in hours slept prior to the study visits, both when comparing saline *vs.* IL-1 antagonism and when comparing lean *vs*. obese subjects. On average lean subjects slept 6.78 ± 0.24 h and the obese subjects 7.04 ± 0.24 h each night three nights prior to the study visits. Daytime sleepiness determined by the Epworth Sleepiness Scale also showed no difference when comparing saline to IL-1Ra and when comparing lean (ESS = 5.38 ± 0.78) *vs.* obese (ESS = 6.13 ± 0.81). Thus, sleep prior to study visits and daytime sleepiness were similar when comparing the two groups and therefore not expected to affect the outcomes.

### 3.3 Role of IL-1 antagonism in postprandial fatigue

**Figure 2:**
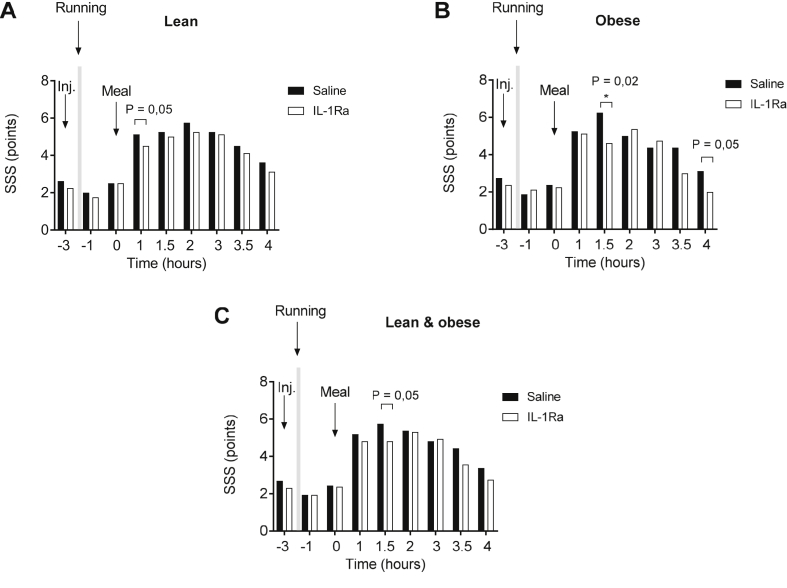
**Postprandial fatigue**. Postprandial fatigue determined at baseline (–3), half an hour after a 30 min exercise bout (indicated by light grey) (–1), and before (0) and after the study meal (1–4) in the lean subjects (**A**), obese subjects (**B**) and lean and obese subjects (**C**) receiving saline (black bars) or IL-1Ra (white bars). SSS = Stanford Sleepiness Scale. * p < 0.05 using a student’s paired *t*-test.

In order to investigate whether postprandial fatigue is driven by IL-1 we injected subcutaneously either saline (placebo) or 100 mg anakinra followed by a high-fat, high-carbohydrate meal [22], [23], [27] to induce postprandial fatigue. Both lean and obese subject's experienced postprandial fatigue as they scored higher on the Stanford Sleepiness Scale already 30 min after the meal was consumed (Figure 2). Comparing the Stanford Sleepiness Scale score in the postprandial period in the presence and absence of IL-1Ra revealed a difference in lean individuals (Figure 2, Panel A) 1 h after the meal was served, whereas in obese subjects anakinra reduced postprandial fatigue 1.5 and 4 h after the meal was served (Figure 2, Panel B). When grouping lean and obese, postprandial fatigue was decreased in the presence of IL-1Ra 1.5 h after the meal was served (Figure 2, Panel C).

### 3.4 CRP and IL-6

Fatigue is associated with an increased level in circulating inflammatory markers. In this study, we measured serum CRP and IL-6 concentrations before and after the intervention (time point −3 and 3 h), thus at baseline and in the afternoon. As expected CRP was increased in obese compared to lean individuals [40]; however, both CRP and IL-6 remained unaffected by the intervention (Table 2).

**Table 2:** **Pre and post intervention measures for Saline *vs*. IL-1Ra in lean and obese**. Data represents mean ± SEM.

	Saline		IL-1Ra	
	Pre	Post	Pre	Post
Lean				
CRP (mg/l)	1.5 ± 0.9	1.3 ± 0.7	2.0 ± 0.9	1.7 ± 0.8
IL-6 (pg/ml)	1.7 ± 0.1	2.2 ± 0.6	1.8 ± 0.2	1.6 ± 0.1
Obese				
CRP (mg/l)	3.3 ± 1.5	3.0 ± 1.4	2.2 ± 0.6	2.2 ± 0.6
IL-6 (pg/ml)	2.0 ± 0.4	2.3 ± 0.2	1.9 ± 0.2	1.7 ± 0.2

## 4 Discussion

The aim of this study was to investigate whether IL-1 regulates postprandial fatigue and whether this is more pronounced in obese subjects. Using a high-fat, high-carbohydrate meal. We induced postprandial fatigue in both lean and obese subjects. The strongest effects of anakinra were detectable 1–1.5 h after the meal, around the time when maximal fatigue was reached. Possibly only at this specific time points the method used was sensitive enough to detect a difference between the treatments.

The fact that the number of hours slept prior to the study visits and daytime sleepiness determined using the Epworth Sleepiness Scale was similar in the two groups indicates that differences in fatigue prior to the interventions did not influence the outcome on study days.

Our study indicates that IL-1 plays a role in postprandial fatigue, but it is probably not the main driver, as all subjects experienced postprandial fatigue even in the presence of anakinra. Another explanation could be the low permeability of the blood brain barrier for anakinra or insufficient dosing [41], [42]. Finally, other cytokines such as TNF have been implicated in fatigue due to diseases [43] and may also play a role in the context of postprandial fatigue.

We combined an acute, moderate bout of exercise with a heavy meal to maximize, in a physiological way, postprandial fatigue. This makes the interpretation somewhat more difficult. However exercise induces only a short-term anti-inflammatory response [44]. Therefore, it is unlikely that it impacted significantly the fatigue assessed 3 h later. Even if the exercise bout would have contributed with an increased level of anti-inflammatory cytokines in the postprandial phase, this would argue against our proposed role for IL-1 in postprandial fatigue.

A causal role for IL-1 in the development of fatigue has been observed in patients with rheumatic arthritis and cryopyrin-associated periodic syndromes; both conditions are characterized by severe fatigue [4], [45]. Furthermore in patients with type 2 diabetes IL-1Ra improves glycemia, insulin secretion [46], and also fatigue [9]. A recent review concludes that inhibition of IL-1 in a broad range of non-inflammatory and inflammatory diseases overall has a positive effect on severe fatigue [2].

The present study indicates that IL-1 is at least partly driving postprandial fatigue in both lean and obese individuals. Thus, patients treated with drugs that antagonize IL-1 could benefit from a reduction in fatigue symptoms in the postprandial state.

## Funding

The Centre for Physical Activity Research is supported by a grant from TrygFonden. The Centre for Physical Activity research is a member of DD2 – the Danish Center for Strategic Research in Type 2 Diabetes (the Danish Council for Strategic Research, grant no. 09-067009 and 09-075724).

Louise Lang Lehrskov was further supported by a grant from Danish Diabetes Academy, which is supported Novo Nordisk Foundation, furthermore Louise received travel grants from the Albert Renold Travel fellowship, Fonden til Laegevidenskabens Fremme and Torben og Alice Fritmodts Fond.

## Author contribution

L.L.L., K.T. and M.Y.D conceived and designed the study. L.L.L, A.M.W, M.H., and J.S. did the medical screened and L.L.L, E.D., and A.M.W performed the experiments. L.L.L. and M.Y.D analysed the data. L.L.L. and M.Y.D interpreted the data. L.L.L. and M.Y.D. wrote the manuscript and all authors contributed and approved the final version of the manuscript.

## Figures and Tables

**Table A.1:** The Stanford Sleepiness Scale (Hoddes E et al., 1973).

Degree of Sleepiness	Scale Rating
Feeling active, vital, alert, or wide awake	1
Functioning at high levels, but not at peak; able to concentrate	2
Awake, but relaxed; responsive but not fully alert	3
Somewhat foggy, let down	4
Foggy; losing interest in remaining awake; slowed down	5
Sleepy, woozy, fighting sleep; prefer to lie down	6
No longer fighting sleep, sleep onset soon; having dream-like thoughts	7
Asleep	X

**Table A.2:** The Epworth Sleepiness Scale (Murray J. et al., 1991).

Situation	Change of dozing (0-3)
Sitting and reading	
Watching TV	
Sitting inactive in a public place (e.g. theater or meeting)	
As a passenger in a car for an hour without a break	
Lying down in the afternoon when the circumstances permit	
Sitting and talking to someone	
Sitting quietly after a lunch without alcohol	
In a car while stopped for a few minutes in traffic	

**Table A.3:** **Study meal**. Nutritional composition.

Main course	Amount(g)	Kcal	Fat(g)	Carbs(g)	Protein(g)
Zopf (bread)	90	269	7.2	41.4	8.1
Butter (Valflora)	15	110	12.3	0.1	0.1
Brie (cheese)	57	190	17.1	0.6	9.1
Walnuts	75	510	47.3	5.3	10.5
Dessert					
Coop chocolate milk	500	325	5	50	17.5
**Total**	**737**	**1404**	**88.9**	**97.5**	**45.3**

## Footnotes

Appendix A Supplementary data related to this article can be found at https://doi.org/10.1016/j.molmet.2018.04.001.
